# Lichens in times of climate change – impacts and responses especially in boreal and polar ecosystems

**DOI:** 10.3897/mycokeys.128.173708

**Published:** 2026-02-03

**Authors:** Lilith Weber, Pekka Niittynen, Annina Kantelinen

**Affiliations:** 1 Botany and Mycology Unit, Finnish Museum of Natural History, University of Helsinki, Helsinki, Finland Faculty of Biological and Environmental Sciences, University of Helsinki Helsinki Finland; 2 Organismal and Evolutionary Biology Research Programme, Faculty of Biological and Environmental Sciences, University of Helsinki, Helsinki, Finland Finnish Museum of Natural History, University of Helsinki Helsinki Finland; 3 Department of Biological and Environmental Science, University of Jyväskylä, Jyväskylä, Finland Department of Biological and Environmental Science, University of Jyväskylä Jyväskylä Finland

**Keywords:** Arctic, biotic interactions, holobiome, snow, soil crusts, species distribution shift

## Abstract

Climate change and biodiversity loss are among the most pressing issues of our time. Lichens have been shown to be sensitive to climate change, but responses are species-specific and contradictory trends have been reported. This review addresses lichen biology in relation to climate change and we overview the responses of lichens (e.g. biotic interactions, species distribution shifts and lichen acclimatisation, adaptation and extinction) to climate (e.g. temperature, precipitation, CO_2_-levels, snow). Research shows mainly adverse or alarming effects of climate change on lichens, but there is not yet a generalisable understanding of the topic. We argue that contradictory trends emerge partly because relatively few studies have been conducted and they encompass a variety of locations, taxa, and methods, which makes them difficult to compare. Moreover, many aspects of lichens are still insufficiently understood, including species diversity, distributions, functional traits and biotic interactions with other organisms. We highlight that future studies would benefit from: 1) Developing a set of model species and also embarking full community studies; 2) Better species data, including monitoring programmes and trait data; 3) Improved conservation planning and Red List evaluations and 4) Acknowledging that lichens are small ecosystems and climate change may affect the partners in ways we do not understand yet.

## Introduction

One of the most pressing issues of our time is the ecological impact of anthropogenic global warming. Total CO_2_ emissions continue to rise, reaching unprecedented heights in 2024 (Shukla et al. 2022; WMO 2025). There has already been a 1.1 °C global temperature increase when comparing the period 2011–2020 with 1850–1900 averages (IPCC 2023). Climate change is predicted to impact a variety of global conditions, from shifting temperatures to changing precipitation patterns, from the frequency of extreme weather events to sea level rise (Shukla et al. 2022). For photoautotroph organisms, this means alterations in growth season conditions (Kong et al. 2017), availability of soil nutrients and nutrient cycling (Nadelhoffer et al. 1991) as well as in habitat availability. These changes have been shown to alter plant and fungal communities and force range shifts (Parmesan 2006; Blois et al. 2013; Newsham et al. 2016; Baldrian et al. 2022). The impacts of climate change are expected to be particularly pronounced in high latitude ecosystems, as the relative rise in temperatures is projected to increase polewards (IPCC 2023). Here, processes related to (perma)frost, glaciers and snow are especially important (Aalto et al. 2014).

**Figure 1.**
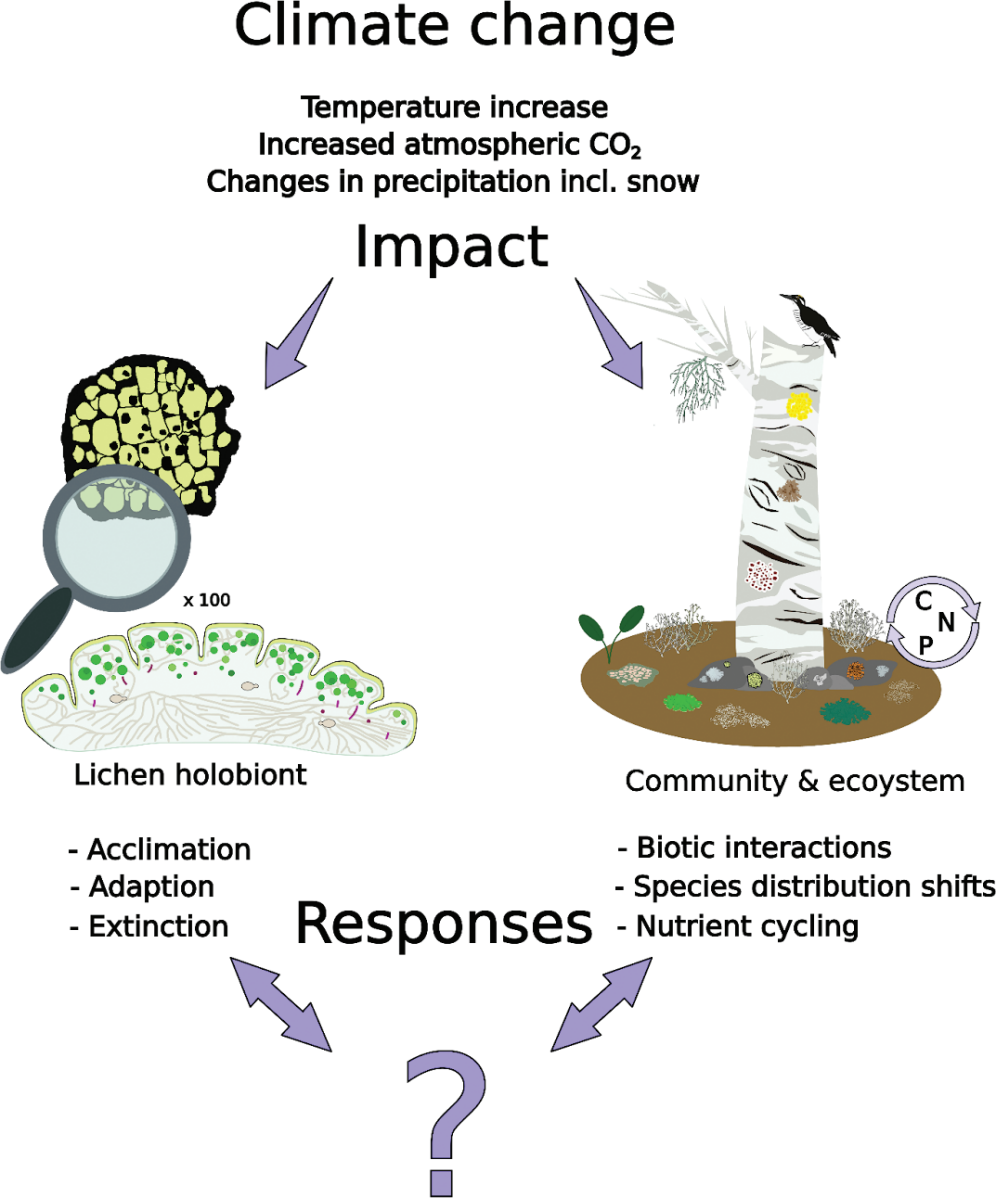
Climate change impacts lichens on holobiont, community and ecosystem levels. The currently known effects are mainly adverse and alarming, but knowledge on the topic is still insufficient and shows some contradictory trends. Lichen diversity, distribution, functional traits and biotic interactions with other organisms are deficiently known.

Symbioses are expected to be particularly susceptible to the effects of climate change, because they are finely-tuned relationships between different partners (Ellis et al. 2007b; Dunn et al. 2009; Nelsen et al. 2022). Lichens — defined as symbiotic associations between one heterotrophic fungus (mycobiont; after which the lichen species is named) one or more photosynthetic algae and/or cyanobacteria (photobiont) and a complex microbiome whose role is still not fully understood (Spribille et al. 2016; Hawksworth and Grube 2020; Spribille et al. 2022; Pichler et al. 2023) — play important ecological roles, such as in soil creation, producing oxygen and fixing nitrogen and providing food and shelter for microbes and animals (Seaward 2008; Elbert et al. 2012; Porada et al. 2018). Despite their reputation as hardy survival specialists, lichens are highly sensitive to changes in temperature and associated environmental factors, which affect their ability to grow and reproduce. Consequently, lichens have been observed to directly respond to climate change (Ellis et al. 2014; Rubio-Salcedo et al. 2017; Mallen-Cooper et al. 2023b; Wrobleski et al. 2023) (Fig. 1). However, the observed lichen responses appear to be species-specific and vary in direction and magnitude between habitats. The range extension of thermophilus, principally (sub-)tropical lichen species into Western and Central Europe, is well documented (Wolfskeel and van Herk 2000; van Herk et al. 2002; Aptroot and van Herk 2007), while in the same geographic areas, remnant populations of formerly more widespread cold-tolerant lichens are declining (Aptroot and van Herk 2007; Hauck 2009). Overwhelmingly, warming has been shown to have negative impacts on species diversity, abundance and growth rates in arid and Mediterranean environments (van Herk et al. 2002; Escolar et al. 2012; Rubio-Salcedo et al. 2017; Temina 2021). Especially in boreal to polar regions, however, contradicting trends are documented, with some species reacting negatively, while others are seemingly unaffected or may benefit from the increased temperatures (Chapin III et al. 1995; Molau and Alatalo 1998; Malmer et al. 2005; Jägerbrand et al. 2009; Hudson and Henry 2010; Lang et al. 2012; Bokhorst et al. 2016; Alatalo et al. 2017; Colesie et al. 2017; Götz et al. 2025).

These species-specific responses of lichens depend on factors such as the presence of different traits, including morphology, substrate preference, dispersal capabilities, photobiont type and bacterial components of the symbiosis (Löbel et al. 2006; Aptroot and van Herk 2007; Rolshausen et al. 2022; Canali et al. 2025). Another factor is the evolutionary legacy of climatic niche evolution of the symbionts (Nelsen et al. 2022). It has been speculated that lineages with slower rates of niche adaptation in their evolutionary past may not be able to adjust to a changing climate as fast when compared to those with a history of evolutionary lability (Holt 1990; Wiens et al. 2010). Within species, genetic diversity might influence their resilience to climate change, with more genetically diverse populations potentially having a higher capacity for adaptation (Colesie et al. 2017).

**Figure 2.**
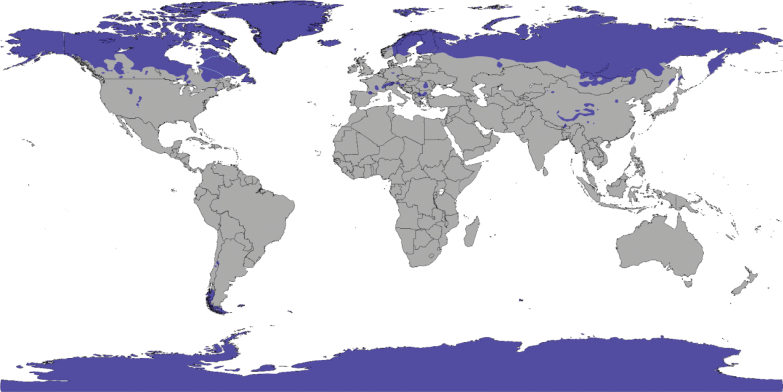
Arctic, alpine and boreal ecosystems globally (Köppen climate classification Dfc, Dwc, Dfd, Dwd and ET, EF (Geiger 1954)).

This review has six sections. In the beginning, we discuss the direct impacts and lichen responses, community- and ecosystem scale factors, and net outcomes. We focus on temperature, precipitation, nutrient availability, snow and ice, biotic interactions, species distribution shifts and how the lichen holobiome can avoid extinction by adapting or acclimatising. We then summarise the topic. At the end, we draw conclusions by identifying research biases and evaluating where additional research could further extend our knowledge. We recommend fruitful avenues for future research. Our aim is to give an overview and synthesise research especially from boreal and polar regions (Fig. 2), with supporting information from temperate regions. We consider both observed responses as well as the results of experimental manipulations to draw general patterns. For simplicity, we did not consider some other phenomena linked to climate change, such as land use change i.e. how humans will adapt the use of land to the climatic conditions. Our approach towards the literature selection was holistic; we did not limit the search to certain search strings such as ‘climate change + lichens’ since this would have excluded valuable research from the past that has much to teach us about the lichen response to climate change, even though ‘climate change’ might not appear in the keywords or title.

## Direct impacts and lichen responses

### Changes in temperature, precipitation and CO_2_-levels

To understand the effect of climate change on lichen growth rates, species distribution and ecological roles, it is critical to consider changes in temperature and water availability and to identify the physiological responses of lichen species to these factors. Lichen growth and survival are directly linked to ambient temperature and moisture, which due to the poikilohydric nature of lichens directly influence thallus water uptake and desiccation (Green et al. 2011; Gauslaa et al. 2012; Merinero et al. 2014). The rates of photosynthesis and respiration increase linearly with increases in temperature up to a certain, species-specific optimum after which these processes are increasingly inhibited (Stocker 1927; Kershaw 1985; Kappen 1993).

Rising temperatures are expected to affect individual growth rates, biomass accumulation, photosynthetic performance, reproductive allocation and carbon balance in lichens (Escolar et al. 2012; Martínez et al. 2012). For example, severe loss of mass and physiological function was found to occur above +2 °C of experimental warming in a decade-long study with the epiphyte *Evernia mesomorpha* by Smith et al. (2018) and Meyer et al. (2023). Additionally, loss of algal symbionts and turnover in algal community composition increased with temperature and were the most apparent impacts of experimental warming. Elevated temperatures also increase drying rates, which can shorten periods of metabolic activity (Kershaw 1985). Temperature and moisture impacts have been found to be the principal factors limiting the position of the warm range edge of species occurrence (Cahill et al. 2014). Extreme temperature increases will likely mean that negative effects on individual thalli will result in changes in the survival, fecundity and growth of populations (Smith et al. 2018; Meyer et al. 2023). These responses also affect species and habitats to different degrees, likely causing differential gains and losses (Ellis et al. 2009).

However, particularly for lichens with cyanobacteria as the photobiont, it has been speculated that there may be benefits from rising temperatures. In general, lichen photobiont net photosynthesis has an optimal temperature, which seems to be species specific. Above this temperature, even moderate increases in respiration will depress net carbon gain and net CO_2_ exchange can become negative (Green and Lange 1994). Cyanobacteria, however, possess a more efficient CO_2_ concentrating mechanism than green algae (Green et al. 1993). This mechanism can slow the increase of photorespiration with higher temperatures by counteracting Rubisco oxygenase activity (Palmqvist et al. 1994). Therefore, the cyanobacterial CO_2_ concentrating mechanism may be advantageous at higher temperatures, where its associated energy cost is lower than the loss associated with photorespiration (Ladón de Guevara et al. 2014). Colesie et al. (2017) speculated that a cyanobacterial photobiont may aid in the adaptation of a lichen to high temperatures, citing studies on tropical and arid lichen species (Wessels and Kappen 1993; Lange et al. 1994, 1998). In their own study in the Antarctic, only *Stereocaulon alpinum*, a tripartite lichen that contains both cyanobacteria and green algae, managed to acclimatise to artificial warming and net photosynthesis did not decrease under a 15 °C warming regime (Colesie et al. 2017).

Whether lichens can benefit from increased CO_2_ availability is as yet unclear (Porada et al. 2022; Stanton et al. 2023). However, limitation of photosynthesis by CO_2_ availability has been demonstrated in lichens, mainly in wet, over-saturated thalli (Palmqvist et al. 2002). The complex interactions of CO_2_, temperature and moisture mean that increased CO_2_ can either promote or inhibit lichen growth (Stanton et al. 2023). Correspondingly, experiments have shown varying effects on different species. In a long-term study on *Evernia mesomorpha* in a boreal forest, enhanced CO_2_ concentrations had no effect on measured ecophysiological traits (Smith et al. 2018; Meyer et al. 2023). In contrast, a study on *Protoparmeliopsis muralis* in a suburban habitat in Germany found an increase in net photosynthesis under certain hydration and light conditions (Lange and Green 2008). It has also been reported that the concentration of CO_2_ is 10% higher in lichen mats and under the thalli of foliose species than in the ambient atmosphere (Tarnawski et al. 1994), possibly because the mats inhibit the diffusion of CO_2_ from the soil.

A field study of lichen photosynthesis rates at Anaktuvuk Pass, Alaska showed that temperatures were not optimal for lichen physiological activity (Nash and Olafsen 2007). In particular, the authors stated that, under 1995 field conditions and under optimal hydration, photosynthesis was primarily light-limited, while nitrogen ﬁxation was temperature-limited in two exemplary cyanolichens. Based on this, they predicted that, if the duration of optimal hydration conditions remains unchanged, then global warming may enhance nitrogen fixation, increasing the vitality of Arctic lichens with cyanobacteria as photobionts. Now, almost 30 years later, it seems that the authors were correct in their prediction that temperatures would increase, while precipitation remained unchanged or increased (Stewart et al. 2022). However, enhanced nitrogen fixation and an increased vitality of Arctic cyanolichens cannot be confirmed. Moreover, significant reductions in nitrogenase capacity at higher temperatures have also been found for representative cyanolichens *Lobaria retigera* in a transplantation experiment (Liu et al. 2018) and *Lobaria oregana* during laboratory experiments (Antoine 2004).

Climate change is already causing an increase in extreme weather events, with the expectation of these becoming more frequent as time goes on (IPCC 2023). These include heat waves and droughts, which lichens, due to their high desiccation tolerance, are better equipped to handle than, for example, most vascular plants. A recent review by Gasulla et al. (2021) describes the various mechanisms that cause the heat and desiccation resistance of lichens, which can remain unharmed at 70–101 °C thallus temperature. In a hydrated and metabolically active state, however, lichens are more sensitive to heat stress, dying when the temperature exceeds 30–48 °C (Lange 1953; Chowaniec et al. 2023). Already in 2024, the Fennoscandian Arctic has seen an unprecedented number of days with maximum temperatures exceeding 25 °C during June and August (Rantanen et al. 2025). Suprasaturation of thalli — an excess of liquid water in the thalli — causes photosynthetic depression regardless of temperature. This is either due to the saturation of the capillary space within the thallus or a formation of a water film on the lichen surface, both limiting the diffusion rate of CO_2_ (Lange et al. 1993).

As net photosynthesis increases linearly up to a certain point with rising temperatures, it is likely that trends in hot deserts will be different from tundra regions. For lichens living in cold regions, temperatures are often not optimal for photosynthesis. The optimal temperatures for studied lichens in these areas appears to be around 10 to 17 °C depending on humidity and radiation (Kappen 1993; Pannewitz et al. 2003; Reiter et al. 2008; Sancho et al. 2019; Chowaniec et al. 2023), but instead, the current mean temperatures in such regions are lower. Experiments show conflicting results. The biomass of soil inhabiting lichens was reported to increase in a short-term warming experiment in Siberia (Biasi et al. 2008), while the ground covered by lichens decreased strongly and consistently over 3 to 4 growing seasons in eleven standardised warming experiments across the tundra biome (Walker et al. 2006). Another study in Sweden and Alaska found that lichen diversity responded negatively to experimental warming over 9 to 13 years in almost all sampled subarctic habitats (Lang et al. 2012). Decreases in lichen coverage were also seen in a 15-year-long warming experiment in arctic regions of Canada (Hudson and Henry 2010). An artificial warming experiment in alpine Norway found that lichen cover decreased significantly (Klanderlund and Totland 2005) and, after 4 years of warming 44% of lichens, had disappeared (Klanderlund 2008). It should be noted, however, that these previously cited studies do not account for competition. As will be discussed in section Biotic interactions amongst lichens, plants and animals, the increased competition from vascular plants that benefit from warming is a major factor impacting lichens. In their study design, Sancho et al. (2019) excluded competition from other organisms, mainly vascular plants and, in that case, found that most lichen species of the Antarctic tundra reacted positively to the warming climate.

Temperature and water availability are closely linked and, therefore, changes in either factor can cause similar lichen responses. This is because heat causes the lichen thalli to dry faster, therefore shortening the photosynthetically active window. Changes in precipitation due to climate change are more variable than changes in temperature and show both positive and negative trends in different parts of the world (IPCC 2023). Forests and the different microhabitats they provide are particularly heterogeneous regarding water availability (Barkman 1958; Porada and Giordani 2021; Di Nuzzo et al. 2022b). For example, lichens in the treetops are affected by rainfall and lichens in shaded canopies experience indirect humidity that is available especially around soft stumps and on the bases of trees. On the other hand, dew is more important in forest gaps and on the bark of tree trunks. Therefore, lichens with widely different moisture optima can co-exist within one site (Root et al. 2014).

Higher temperatures have negative impacts on respiration rates and cause desiccation, but it appears that some lichens have a protective mechanism, for example certain phenols, non-reducing sugars and proteins (Kranner et al. 2008). Pigments, such as the carotenoid zeaxanthin, are formed by lichens to protect chlorophyll during the desiccation process and to promote acclimatisation during rehydration (Adams et al. 1993; Kranner et al. 2009; Fernández-Marín et al. 2010; Ndhlovu et al. 2024). A study by Fernández-Marín et al. (2010) suggests that, if desiccation occurs faster than normal, photoprotective mechanisms fail, leading to structural damage.

It is also essential to consider certain lichen traits when discussing the impact of temperature and water availability. Foliose and fruticose growth forms are exposed to changes in air temperature and humidity to a greater degree than crustose lichens due to their higher surface-to-volume ratio (Larson 1984; Esseen et al. 2015). Crustose lichens are also more closely attached to their substrate, which will often have a greater water holding capacity and moderate changes in temperature. The presence of a cortex and thallus surface properties such as hydrophobicity are also traits that influence thallus (de)hydration and, therefore, shape the ecological niche of different lichen species (Lakatos et al. 2006; Gauslaa 2014; Canali et al. 2025).

### Changes in snow and ice

Snow, which is an important feature of high latitude and altitude biomes, is also changing greatly under conditions of climate change. The predicted changes in climate over the next 50 years are expected to be most pronounced in Arctic and subarctic regions (IPCC 2023). During the last decades, the Arctic has already warmed almost four times faster than the global average (You et al. 2021). The strongest recent warming has occurred in autumn and winter months with significant consequences for the extent and quality of snow and ice (Kausrud et al. 2008; Chen et al. 2011; Bitanja and van der Linden 2013; You et al. 2021). Increased winter temperatures have led, amongst other things, to an increased frequency of winter thaw and ground-icing events (Cooper 2014).

Snow affects organisms in a multitude of ways. The microenvironment beneath the snow is insulated from the elements and protected from grazing (Edwards et al. 2007). However, a thick snowpack reduces the rate of photosynthesis beneath it and severely shortens the growing period. Another factor is the availability of moisture, as snow beds have a high input of meltwater, even during late summer droughts (Niittynen et al. 2018). While there is still much more research needed, we do know that snow has a strong effect on lichen communities (Sonesson et al. 1994; Bidussi et al. 2016; Niittynen et al. 2018; Odland et al. 2018). Studies on vascular plants, mosses and macrolichens indicate that a 20–30% reduction in snow cover significantly increases the probability of endangerment and even extinction of the associated species (Niittynen et al. 2018). The significance of snow for the future of mountain species is suspected to be even greater than the direct effects of rising temperatures (Niittynen and Luoto 2018; Niittynen et al. 2020). Some lichens are extremely freeze-tolerant in a dry state (Kappen 1993) and can be photosynthetically active at very low temperatures such as -20 °C, as in the case of *Cetraria nivalis* in northern Finland (Kallio and Heinonen 1971). Some lichens can be photosynthetically active also under snow by absorbing water from high air humidity (Pannewitz et al. 2003). It has been shown that, for sub-arctic *Cladonia* species, photosynthetic performance was significantly lower in species that were not insulated by snow (Bjerke 2009), confirming the protective qualities of snow cover. Ground icing following partial snow-melt or rain-on-snow events, however, have been shown to be detrimental to lichens. Ice-encapsulation for more extended periods of time at mild subfreezing temperatures can be lethal to the same *Cladonia* species, as well as to other groups, including epiphytic lichens (Benedict 1990; Bjerke 2011). However, it should be noted that Finne et al. (2025) found that their studied lichens (*Cetraria islandica*, *Cladonia mitis* and *Foveolaria nivalis*) possess a stronger tolerance against thaw–freeze and ice encasement than some co-existing plants. A similar trend was shown by Sundstøl and Odland (2017). While some boreal-arctic lichens are highly adapted to mild subfreezing temperatures under a thick layer of snow (MacFarlane and Kershaw 1980; Scott and Larson 1985, 1986), species from higher latitudes where warm winters have, until recently, been rare, might be incapable of surviving the hypoxic conditions that ice encapsulation brings, especially during the long, dark winter at high latitudes (Bjerke 2011). If freezing constraints would be lower, then mid-elevation and temperate species might expand their distribution ranges; this might increase competitive pressure on current high-latitude/altitude lichen species (Worthy et al. 2024).

In addition to the seasonal snow, the widespread melting of glaciers may result in new habitats becoming available in high latitude and altitude biomes (e.g. Singh and Ravindra (2012); Halda et al. (2025)). This would mainly benefit stone-inhabiting and pioneer lichen species. However, lichen habitats would also be likely lost when vascular plants invade new vegetation-free areas (Fraser et al. 2014; Barredo et al. 2020).

The melting ice also causes sea levels to rise globally. This leads to the erosion of key coastal habitats, shifting nutrient availability and increasing the frequency of floods (Passeri et al. 2015). It is estimated that, by the year 2100 in boreal to polar regions, sea level rise will be between 0.25 m (Finland) up to a maximum of 0.78 m (Russia), based on the high emissions SSP5-8.5 Shared Socioeconomic Pathway scenario (IPCC 2023). Most land that will be below the tideline in this scenario will be in the Northern Hemisphere i.e. especially parts of the coasts of Alaska (USA), Yukon (Canada), Nenets and Yakutia (Russian Federation) (Vanderlinden et al. 2018; Kulp and Strauss 2019; IPCC 2023; Climate Central 2025). Several studies have shown that sea level rise can be one of the most important long-term threats for coastal lichens (Lendemer and Allen 2014; Allen and Lendemer 2016b; McMullin et al. 2019).

### Excursus: Climate change affects biological soil crusts

A significant number of studies on lichens and climate change have focused on biological soil crusts and their specific response to climate change (e.g. Walker et al. (2006); Biasi et al. (2008); Maestre et al. (2013); Li et al. (2020); Mallen-Cooper et al. (2023a); Wang et al. (2024)). Hence, we give an exemplary research overview of the topic. Biological soil crusts (BSC) are microecosystems in which the top few millimetres of soil particles are connected and stabilised by soil-inhabiting organisms, such as cyanobacteria, algae, fungi, lichens and mosses. Lichens especially dominate in late successional stages of biocrust development (Rosentreter et al. 2016). When only considering high latitudes, biocrusts are especially common in Patagonia, Siberia, northern North America and coastal areas of Greenland (Wang et al. 2024). Habitats where BSC are most prominent, such as hot and cold deserts, drylands and polar regions (Belnap et al. 2001; Belnap and Lange 2003), are also suggested to be amongst the first and most severely affected by the predicted increase in temperature (IPCC 2023). Multiple studies (Evans et al. 2001; Escolar et al. 2012; Maestre et al. 2013) predict that global warming might have a disastrous effect on BSC, especially in arid areas.

BSC organisms are largely poikilohydric, meaning their hydration and metabolism are highly dependent on ambient moisture and temperature (Ladón de Guevara et al. 2014). Warming increases the saturating water capacity of air, which leads to greater drying stress (Campbell and Norman 1998). Studies have shown that changes in rainfall, fog or dew and temperature are likely to affect the functioning and community dynamics of BSC. Rain frequency and duration of dry periods have been found to be key factors controlling the development and composition of BSC in two dryland study areas in south-western Africa (Büdel et al. 2009) and the south-western USA (Evans et al. 2001; Belnap et al. 2004; Reed et al. 2012; Zelikova et al. 2012). Areas of higher precipitation and lower temperatures are positively correlated with higher prevalence of lichens in BSC (Rogers 1972). A study in Spain (Escolar et al. 2012) that investigated the effects of artificial warming of up to 2.4 °C within 3 years showed a dramatic decrease in lichen coverage in the established BSC plots and a notable decrease in the lichen colonisation of bare ground. This response was not regulated by changes in precipitation. These results are in line with other artificial warming studies of 2.1–3.8 °C, in the US (Belnap et al. 2006), central Spain (Maestre et al. 2013) and arid South African ecosystems (Maphangwa et al. 2011). However, in contrast, a 12-year-long study of experimental warming between 0.5 °C and 1.5 °C in the Tengger Desert, China, showed that lichens were not sensitive (Li et al. 2020). These latter experimental warming estimates may have been too conservative to accurately predict future developments, as temperatures exceeded the global 1.5 °C threshold in January 2024 (Copernicus Climate Change Service 2021; United Nations Environment Programme 2025).

## Community- and ecosystem-scale factors

### Biotic interactions amongst lichens, plants and animals

Theory suggests that as climate warms, the most dramatic effects will come from changes in species interactions and community assembly, rather than from the direct effect of limited physiological tolerance (Urban et al. 2012; Blois et al. 2013; Cahill et al. 2014; Ockendon et al. 2014). These indirect effects occur because of a change in the performance and abundance of competing species and, in the long term, also a change in competitors’ identity caused by local extinctions and migration. These novel species assemblages might be interacting in still undiscovered ways (Gilman et al. 2010).

One example of such interactions is the advancing treeline in high altitude and latitude environments. In this case, a key question is whether the epiphytic lichen communities are able to keep up with the range shifts of their habitat. Many forest-inhabiting lichens will likely be significantly affected by changes in forest range, for example, because they require a forest structure associated with long continuity (e.g. Rose (1976); Rose and Wolseley (1984); Paillet et al. (2010); Saine et al. (2018)), such as damp, shaded conditions, presence of dead wood and rough bark characteristic of old trees (e.g. Barkman (1958); Hyvärinen et al. (1992); Holien (1996); Fritz et al. (2009); Ranius et al. (2008); Nascimbene et al. (2009)). A study by Greenwood et al. (2016) suggests that the woody species that advance to arctic tundra – where previously no trees occurred – can have permanent negative impacts on species richness. However, in other rapidly advancing treelines, the negative impacts may be less drastic. This is because outside of the treeless tundra, the advancing woody species are mainly altering stand dynamics inside already established forests, instead of spreading into treeless habitats. For example in subarctic Fennoscandia, conifers are gradually replacing the typical mountain birches (Kauhanen 2013; Niittynen et al. 2020).

In addition to lichens that grow on trees, changes in vegetation will affect areas that have currently only few vascular plants. Terricolous lichens are a widespread and ecologically important component of high altitude and latitude soil ecosystems (Matveyeva and Chernov 2019). Their local abundance is explained by the small size of the vascular plants (Cornelissen et al. 2001; Odland et al. 2018); the harsh climatic conditions in high altitude and latitude environments and limited availability of soil nutrients means that vascular plant growth is reduced and, therefore, lichens can compete successfully. The latitudinal and altitudinal progression of the tree line and the spread of shrubs into mountain heaths means that the habitat of many species that depend on open fells – including terricolous lichens – is shrinking (Myers-Smith et al. 2011; Xu et al. 2013). A change towards more shrubs and trees would cause significant shifts in terms of lichen competition for space, light and nutrients. It would also change the conditions on the ground: forests are generally more humid, have deeper snowpacks and more physical cover from leaf litter (Cornelissen et al. 2001; Roth and Nolin 2017). Various studies addressing the influence of increasing temperatures in the arctic tundra show that terricolous lichens are at a disadvantage compared to vascular plants (Chapin III et al. 1995; Cornelissen et al. 2001; Epstein et al. 2004; Hollister et al. 2005; Walker et al. 2006). Likewise, bryophytes in tundra systems have been shown to outcompete lichens under conditions of climate change (Vanneste et al. 2017; Vuorinen et al. 2017), while Schwager et al. (2024) and Mayo de la Iglesia et al. (2024) predict future climate to favour lichens over bryophytes due to reduced humidity stability in alpine grasslands and lower alpine summits (see also Michelsen et al. (2011)). Depending on the severity of warming and the timeline considered, climate change will likely affect lichens first in the climatically milder parts of the Arctic. Cornelissen et al. (2001) hypothesised that, in these milder parts, where ecosystems are characterised by relatively dense plant canopies, climate warming and increased nutrient availability would lead to a decline in macrolichen abundance due to increased vascular plant biomass. This hypothesis was supported by data from ecosystem manipulation experiments in Svalbard, Alaska, Canada and Sweden conducted between 66 and 84 °N. The data, collected over 3 to 10 growing seasons with active or passive warming of 2.5 to 5 °C, showed consistently negative relationships between the abundance of macrolichens and vascular plants. However, this pattern was not as clear in the coldest high-arctic or arctic-alpine sites. What should be kept in mind, however, is that these responses will not be uniform across the high latitude and altitude biomes. Saxicolous and epiphytic lichens or terricolous lichens at very dry exposed sites are subject to very limited competition with vascular plants, so direct temperature impacts may be more relevant (Cornelissen et al. 2001; Čabrajić 2009; Mayo de la Iglesia et al. 2024).

Many lichens are specialised to grow on certain phorophytes because of the different chemical and physical features of bark and wood amongst contrasting tree species (Ódor et al. 2013; Benítez et al. 2019). Tree species are expected to respond to climate change, though the extent of this effect and trends are different depending on tree species and region. Range shifts, expansions, contractions and also extinctions are expected for different tree species, depending on their tolerances for heat and drought, life cycle speed, evolutionary histories and dispersal mechanism (Kirschbaum 2000; Dyderski et al. 2017; Fei et al. 2017). All of these outcomes are expected to take effect rather slowly for trees, with range shifts in the scope of a few metres per year or some tens of kilometres per century (Iverson et al. 2004; Feeley et al. 2012; Lazarus and McGill 2014). At this pace, it seems likely lichens will not be left behind as phorophyte distribution changes (Root et al. 2014), although other essential forest features might be lacking at the leading edge, like dead wood diversity including weathered old snags and heavily decayed large stumps, (fire)scars on trees within a closed canopy, rough bark and hollows etc., since it takes a long time for trees to develop these characteristics (Rose and Wolseley 1984; Nordén et al. 2014; Saine et al. 2018).

In some cases, however, extinctions caused by climate change are happening fast. In many areas a shift in wildfire regimes towards more frequent and/or more severe fires has been observed (Moritz et al. 2012). Lichens are known to be generally sensitive to fire and both terrestrial and epiphytic communities take decades to recover from fire disturbance (Greuel et al. 2021; Miller et al. 2021). Under climate change, fires may occur too frequently and have too severe of an impact on ecosystems for lichens to regenerate, causing broad and persistent landscape-scale losses (Miller et al. 2018). In addition to the lichens themselves being harmed, wildfires can also cause the (local) extinction of host tree species that are an obligatory substrate for certain lichen species (Davis et al. 2019).

Climate change is reshaping lichen communities through multiple animal-driven processes. Reindeer and caribou, for example, are highly sensitive to snow conditions and adjust their lichen foraging accordingly (e.g. Helle and Tarvainen (1984); Roturier and Roué (2009); Stien et al. (2010)), while high-altitude grazers, such as chamois, ibex and Dall sheep, are expected to experience shifts in population and feeding patterns (Sägesser and Krapp 1986; Ivanenko et al. 2004; Deutz 2019; Lambert Koizumi and Derocher 2019; Nozières 2019). In Patagonia’s steppes, increasing aridity has intensified the damage that grazing livestock inflict on BSC, including lichens (Yahdjian et al. 2023). Other herbivores also play a role: warmer and shorter winters extend the grazing season for gastropods, which is particularly relevant in temperate and boreal zones (Asplund et al. 2009; Gauslaa et al. 2018). The chance of massive bark beetle outbreaks are also increasing (Seidl and Rammer 2016) because of more severe storms (Bengtsson et al. 2008; Bhatia et al. 2019). Storms and bark beetles affect lichens by destroying the thalli of macrolichens and damaging forest habitats (Esseen et al. 2022). Finally, changes in bird migration, such as shorter distances or even year-round residency in high-latitude breeding grounds (Lawrence et al. 2021; Kubelka et al. 2022), may disrupt bird-mediated dispersal of lichens (Johansson et al. 2025).

### Changes in nutrient cycling

Global warming also causes rising soil temperatures, leading to faster nutrient cycling and mineralisation (Dai et al. 2020). In general, higher temperatures cause an accelerated terrestrial carbon and nitrogen cycle, while the impact on phosphorus is less well understood (Margalef et al. 2021; Sun et al. 2022). In high latitude and altitude environments, this also means shrinking of permafrost and deepening of the active layer of soils. This will allow nutrient release from decomposed organic soil matter (Jin et al. 2021). Lichens dominate many ecosystems that are poor in nutrients and, therefore, not accessible to most vascular plants. Enhancement of nutrients because of rising soil temperatures can be expected to have a significant impact on lichens and their interactions with vascular plants.

Experimental nutrient enhancement (nitrogen, phosphorus and potassium) by Press et al. (1998) in a subarctic shrub heath at Abisko, northern Sweden, caused the decline of mosses and lichens. On the contrary, manipulations in Antarctica have found increased productivity in lichen-dominated communities over two years (Wasley et al. 2006).

### Excursus lichens in peat bogs

Lichens are also prominent components of bogs and peatland, especially the dry and raised microtopography (Ahti and Oksanen 1990; Harris et al. 2018). This habitat is responding notably to climate change. In Europe, peatlands have undergone substantial, widespread drying (Swindles et al. 2019), but permafrost peatlands exhibit both wetting and drying under climate change (Sim et al. 2021). Temporarily, bog drying might favour lichens, but in the long-term, dry bogs are overgrown by trees and other tall vascular plants, suppressing lichen growth (Rydin and Jeglum 2006).

## Net outcomes

### Species distribution shifts

Climate is one of the key factors determining the distribution of species (MacArthur 1984; Pearson and Dawson 2003). Many species that are exposed to a warming climate respond by migrating or otherwise shifting their distribution. Cold-temperate species might change their spatial distribution to cooler climates, towards higher elevations or polewards (Parmesan 2006; Lenoir and Svenning 2015; Allen and Lendemer 2016a; Mayo de la Iglesia 2024; Götz et al. 2025; Francesconi et al. 2025). In contrast, the distribution of warm-temperate species might become broader as a result of the higher availability of suitable thermal habitats (Rubio-Salcedo et al. 2017).

Many lichen species are dispersed widely and can colonise new areas relatively fast. Due to this trait, pronounced shifts in distribution have been predicted, based on habitat preferences and climate change projections (Ellis et al. 2007b). In Sweden, north-north-east shifts of *Hypogymnia physodes* and *Vulpicida pinastri* have been attributed to changes in temperature and precipitation (Lättman et al. 2009).

In temperate areas, distribution shifts and colonisations of warmth-adapted lichen species have already been observed (Wolfskeel and van Herk 2000; van Herk et al. 2002; Aptroot and van Herk 2007). One of the studies (van Herk et al. 2002) had focused on exposed roadside trees because an original intention was to document changes resulting from air pollution levels i.e. sulphur dioxide and ammonia. These factors had clear effects as the prevalence of pollutants changed. However, shifts in species presence from 1995–2001 could not be explained only by air pollution variables or nutrient availability, but instead, such shifts showed a positive correlation with temperature and oceanity. The authors showed that warmth-loving, oceanic lichens are expanding and boreal lichens are decreasing. Eventually, warming climates will likely cause local extinctions of cold-adapted species, while warm-adapted species will increase – this process is known as “thermophilisation” (Vanneste et al. 2017).

A similar effect has also been shown in nearby countries: Belgium (Broeck 2010), Denmark (Søchting 2004) and Germany (Stapper et al. 2011; Kirschbaum et al. 2012; Windisch et al. 2012; Stapper 2012; Stapper and John 2015; Dittrich 2022; Wutz and Diekmann 2025). In Germany, a list of 45 epiphytic climate change indicator species and measuring guidelines to assess local climate has been developed (VDI 2017) and is in use in the public monitoring programmes of some regions (Stapper 2017; Eichler et al. 2019). Alternatively, the “Species Temperature Index” following Sparrius et al. (2018) can be used to monitor possible shifts in local species composition towards more thermophilic species. When monitored according to the VDI protocol, the mean number of these indicator species has increased significantly (Franzen et al. 2002; Stapper and Franzen-Reuter 2018; Stapper 2023). However, the indicator species lists should be used with caution, as they are based on a comparably small number of (historical) records. An evaluation by Nelsen and Lumbsch (2020) found that the majority of species designated as indicators of climate change in the German indicator list (VDI 2017) had either insufficient data for evaluation or have not exhibited a range shift consistent with that expected under climate change. In addition, there have been other attempts to establish lichens as climate change indicators in different ecosystems. For example, Root et al. (2014) proposed twelve macrolichens that they found to be strongly related to climate variables for southeast and south-central Alaska.

Species distribution modelling is an approach that has been frequently used to predict how climate change will impact biodiversity. There have been more than 34 studies focused on modelling lichen distributions under climate change, mostly from temperate regions (see, for example, Ellis (2019a); Mallen-Cooper et al. (2023a)). Most recently, Mallen-Cooper et al. (2023a) undertook global modelling of eight ecologically significant and well-recorded lichens from temperate and Arctic regions. The suitable climatic space for five lichen species (i.e. *Cladonia stellaris*, *Nephroma arcticum*, *Nephromopsis nivalis*, *Peltula patellata* and *Stereocaulon paschale*) was predicted to expand, while the space for two species (*Collema coccophorum* and *Placidium squamulosum*) were predicted to contract. The study acknowledged, however, that it is unclear if the necessary speed of migration is realistic and if the land use and novel biotic interactions in areas of newly-available climate space would allow successful establishment.

Rubio-Salcedo et al. (2017) predicted a loss of suitable climate space by the 2080s for 75% of the 41 species they modelled in the Iberian Peninsula. They found that the predicted effects were trait-related and connected to lichen growth form, photobionts and substrata preferences. Results indicated that macrolichens, especially those with cyanobacteria as a photobiont, are affected most negatively. On the other hand, crustose lichens with green-algae as a photobiont, were predicted to be more resilient or even benefit from climate change. Saxicolous and epiphytic species were also predicted to be resilient. Their models indicated range shifts for all of the studied species.

A study by Ellis et al. (2007b) predicted the response of 26 lichen species representing five biogeographic patterns in Britain, using 10 km grid-squares and data from the British Lichen Society mapping scheme collected since the 1960s. They found a consistent and strong decrease of suitable climatic space for species characterised as Northern-Montane and Northern-Boreal and a consistent increase for Southern-widespread species by the 2050s. These results are, however, in conflict with the modelled future climate space of a single species *Myriolecis populicola*, which was analysed separately by Ellis et al. (2007a). *Myriolecis populicola* occurs currently in north-east Scotland and it was chosen because the authors had gathered an exceptionally high-quality presence/absence dataset of this species. It was considered suitable for the study also because it is probably not dispersal limited and not constricted by habitat resources and, therefore, its range is mainly limited by climatic variables. The suitable climate space for *Myriolecis populicola* was predicted to increase throughout most of unpolluted northern Britain by 2050, with a shift towards more continental and dry parts of eastern Scotland. The authors took this result as a warning that opposing predictions may suggest there could be future non-analogue lichen assemblages i.e. species that at present inhabit similar geographic ranges, but respond differently. Later, Ellis et al. (2014, 2015) focused on epiphytic lichens in Britain and found that, for 382 species in a high emissions scenario (SSPC 5-8.5, see IPCC (2023)), 38% would lose suitable climate space and 62% gain suitable climate space by the 2080s. In contrast to previous studies, the taxon selection was not focused on species expected to be sensitive to changes in climate, but instead used all epiphytes with sufficient records on a range of selected tree hosts. This might explain the generally more positive response to climate change.

When modelling species shifts, it is important to consider the environmental variables as completely as possible. A study by Crabtree and Ellis (2010) in the Scottish mountains showed that approaches that only considered factors of temperature (or altitude as a proxy) would have indicated an upward shift in montane vegetation zones and, therefore, a decline in montane lichen occurrence at lower to mid-altitudes and also possibly an increase at higher altitudes in different climate change scenarios. However, when incorporating wind speed, the modelled response changed fundamentally. In the case of decreasing wind speeds, as is projected for Europe according to the CIMP6 climate projections (Copernicus Climate Change Service 2021), lichen occurrence would be more severely reduced over a larger range of altitudes, due to increased canopy height and competition.

In general, the quality of models predicting the future species distributions depends on the accurate characterisation of the factors shaping current distributions. Including factors such as biotic interactions, pollution, wind speed, irradiance or snow that are important for species’ functional ecology have been found to be critical for creating more realistic predictions of how communities will develop under climate change (Ellis et al. 2007a; Mod et al. 2016; Ellis et al. 2017; Niittynen and Luoto 2018; Ellis 2019a). Especially for lichens, the resolution of variables is crucial. Lichen presence is highly dependent on microclimatic factors, which can be overlooked at the scale usually used for species distribution models (Ellis et al. 2017; Ellis 2019; Porada et al. 2022). Microclimatic refugia can allow lichens to remain *in situ* by maintaining suitable conditions of water and temperature (Ellis 2020; Di Nuzzo et al. 2022b). The importance of these microrefugia is perhaps greatest in forests, where canopy and bark structures create small-scale heterogeneity of light, wind and precipitation (Porada and Giordani 2021; Di Nuzzio et al. 2022b).

Estimations of potential range shifts vary between species and regions. Mallen-Cooper et al. (2023a) estimated that an average range shift of 50 km per decade by 2100 would be required for eight lichen species globally based on data deposited in GBIF. On the other hand, for epiphytic lichens in Scotland, an average of 60 km per decade was estimated by the 2080s (Ellis and Eaton 2016). While evidence for range shifts due to climate change are still rare, such shifts have been observed with lichens recolonising into areas where they were previously lost due to air pollution (e.g. Rose and Hawksworth (1981); Weldon and Gradin (2021); Paoli et al. (2021); Baumann et al. (2022)). However, these observed range shifts have been comparatively slow, for example ca. 18 km per decade in London (Hawksworth and McManus 1989) or 4–6 years for ca. 1 km in Leoben-Hinterberg, Austria (Hafellner and Grill 1980). In general, effective dispersal rates are closer to 10s to 100s of metres per decade, with the exception of rare long distance dispersal events (Belinchón et al. 2017; Roturier et al. 2024), though studies on the speed of not only dispersal, but also establishment and time until reproduction are scarce. Overall, it therefore seems unlikely that natural migration would be sufficiently fast enough to track the suitable climatic spaces. One solution would be assisted migration, which however, so far, has only been attempted with a few select, large lichen species (Hallingbäck 1990; Gilbert 2002; Brooker et al. 2011; Allen 2017; Brooker et al. 2018; Mallen-Cooper and Cornwell 2020; Balderas et al. 2025). Additionally, there are many uncertainties regarding assisted migration, from legal and practical aspects to the unknown impact that these transplants have on their new ecosystem (Smith 2014).

In mountain ecosystems, range shifts are and will happen towards greater elevation. Vallese et al. (2021), for example, suggested that distributions of *Peltigera* species in the European Alps will contract towards the inner, higher Alps by 2100. A similar trend was predicted for *Lobaria pindarensis* in the Himalaya (Devkota et al. 2019). The situation for lichens confined to high montane areas can appear dire, as they may be isolated from the next suitable habitat (Aptroot et al. 2016). The situation is comparable for arctic island species. Rare and endemic species are also highly threatened in more well-connected areas, such as the Appalachians, as was shown by Allen and Lendemer (2016a), who predicted more than 90% loss of suitable climate space for high-elevation Appalachian lichen endemics. These studies contribute to the now commonly accepted view that climatic vulnerability is greatest amongst high-elevation and high-latitude communities. In contrast, a study by Smith et al. (2020) found that the most vulnerable communities are in low-elevation and southerly locations. They focused on North America, using a large herbarium-based dataset of 172,127 unique occurrences of 443 epiphytic macrolichens species at 46,343 sites. To account for the unequal sampling efforts that go into herbarium records, they used coarse-grained grid cells of 50 km × 50 km, calculating the mean climate value per grid cell. They found that at least one-fifth of all U.S. epiphytic macrolichen communities would face a severe loss of species by the 2070s in a high-emissions scenario. It should be noted, however, that epiphytes are already rare or absent in the hottest regions and in the highest elevations of the studied area, corresponding to habitats above the tree line or in deserts (Hansen et al. 2013).

Species distribution modelling is one of the most powerful tools to understand the influence of climate change on species’ ranges, despite the fact that the modelled shifts only represent shifts in the location of suitable habitat and not of materialised species’ presence, which will depend on several factors besides climatic suitability (such a dispersal, competition, habitat quality etc.) (for a detailed overview, see Ellis (2019a)). However, when attempting to model species future distributions, one major issue is the uncertainty regarding the species’ realised niche, which arises from incomplete or biased datasets of current presence and absence of species. While presence data for lichens is usually fairly reliable, the quality of absence records is much weaker, especially across large study areas. Many species are easily overlooked or require specialised knowledge or equipment to be identified. However, even common species and easily identified species suffer from false absences, since they can be perceived as uninteresting and will, therefore, remain under-reported. As a result, models risk underestimating the true range of species, leaving gaps in our understanding of their ecological niches. Great care should, therefore, be taken to use lichen occurrence data with a suitable degree of completeness for the scale of analysis (for example, Ellis et al. (2007a, 2009); Francesconi et al. (2025)).

### Acclimatisation, adaption and extinction of the lichen holobiome

Some authors have argued that it is essential to focus mainly on distribution shifts and extinction as responses to climate change, because rates of evolutionary adaptation cannot hold up with the speed of recent and future climate change (Parmesan 2006; Jezkova and Wiens 2016; Smith et al. 2020). These studies have, however, treated the lichen as a ‘single species’ in considering niche adaptation. In contrast, lichens may be better considered as a combination of different taxa that form one holobiont. In such symbioses, the climate sensitivity depends on the responses of individual symbionts, as well as the aggregate association – and the responses of the partners are often asymmetrical (Kiers et al. 2010; Colesie et al. 2017). When referring to the extinction of a lichen species, what is typically meant is the extinction of the lichenised fungus. One such fungus can associate with different algal and/or cyanobacterial species and strains and this is what is often considered as a single lichen. However, it is known that co-existing algal strains differ in their physiological tolerances (Casano et al. 2011; Pérez-Ortega et al. 2023). Hence, lichens have a potential mechanism to acclimatise in a changing environment: the switching of photobionts.

Photobiont performance and temperature sensitivity are speculated to be the basis for observed negative outcomes in terms of species number and lichen cover under climate change in temperate and Mediterranean ecosystems (Aptroot and van Herk 2007; Escolar et al. 2012; Rubio-Salcedo et al. 2017; Smith et al. 2018) due to chlorophyll degradation and photobiont loss (Pisani et al. 2007; Meyer et al. 2023). The importance of photobionts has already been partly addressed above, but can also be inferred from present-day changes in distribution. In the Netherlands, a shift toward lichens with the green algae *Trentepohlia* as the photobiont can be observed. Lichens with *Trentepohlia* are more sensitive to frost than, for example, those with *Trebouxia*, but are able to utilise water vapour more efficiently (Nash et al. 1987). Their respective mycobionts belong to unrelated taxonomic groups and prefer different habitats, but they share a southerly distribution in their biogeography (Aptroot and van Herk 2007; van Herk 2009). It is argued that this observed distribution and the parallel species shift under climate change is directly related to photobiont algal ecology.

The *Trentepohlia*-associated mycobiont species belong to a given lichen guild, meaning that several different mycobionts can associate with the same algal species (Rikkinen et al. 2002). Conversely, one mycobiont can also associate with multiple genera, species or strains of photobiont algae (Blaha et al. 2006; Singh et al. 2016; Williams et al. 2017; Ertz et al. 2018; Dal Forno et al. 2021), either exclusively or simultaneously in the same thallus (Casano et al. 2011). This is possible by recruitment of free living algae (Sanders and Masumoto 2021) or by horizontal transfer between holobionts (Nelsen and Gargas 2008; Dal Grande et al. 2012). The mycobiont may, therefore, be able to ‘select’ photobionts that are locally adapted to optimise their ﬁtness in a given habitat or climate (Osyczka et al. 2021). This might be an opportunity to acclimatise to climate change, as has been implied by studies observing a turnover of photobionts along elevation gradients (Dal Grande et al. 2018; Rolshausen et al. 2020, 2022; Medeiros et al. 2021). However, it is not yet clear if acclimatisation by recruiting new symbiotic partners is an option for established thalli. An experimental study using the soil-crust lichen *Psora decipiens* found that the globally differing fungal genotypes have a high degree of speciﬁcity for a narrow range of photobionts and the species was unable to acclimatise by photobiont selection to the substantial climatic variability across its environmental range (Williams et al. 2017). Another study on the cosmopolitan lichen genus *Protoparmelia* and the associated *Trebouxia* algae showed that the frequency of photobiont switches and specialisation depends on evolutionary history and habitat, with switches being more common in warm climates (Singh et al. 2016).

For photobiont switches to be a successful acclimatisation strategy, suitable photobionts would need to be present within the range of the mycobiont. However, according to a recent study by Nelsen et al. (2022), the photobionts of more than 7000 lichens that have an algae belonging to the family *Trebouxia*, cannot adapt to temperature changes as quickly as the Earth is warming. The study compared the climatic preferences of different *Trebouxia* species and their evolutionary history, using phylogenetic analysis and statistical models. The results showed that *Trebouxia* has diversified into different lineages that occupy different climatic niches, ranging from tropical to polar regions. However, these lineages have evolved slowly over millions of years. The predicted rate of modern climate change vastly exceeds the rate at which their thermal tolerance or occupation has historically evolved and, therefore, they may not be able to evolve to cope with the rapid pace of climate change that is expected to occur in the next century. The authors concluded that certain parts of the current range of *Trebouxia* are likely to become inhospitable to them.

In addition to the algae (and possibly cyanobacteria), non-photosynthesising bacterial components of the holobiont might also have the potential to facilitate acclimatisation (Spribille et al. 2022). Rolshausen et al. (2022) found a predictable turnover of bacteria with altitude, in their experiment on *Umbilicaria* in Spain. Klarenberg et al. (2020) were able to demonstrate a shift in the bacterial community composition of *Cetraria islandica*, comparing thalli that underwent 20 years of warming to a control. The warmed plots had a temperature increase of 1–2 °C and also had an increase in dwarf-shrub and litter abundance, realistic effects of climate change in low arctic *Betula nana* heath. In a study on the microbiome of *Lobaria pulmonaria* along a north-south transect of ca. 1100 km in central to northern Europe, climatic variables explained 41.64% of microbiome variation (Bogale et al. 2024).

Lastly, the ability of the fungal partner to acclimatise through phenotypic plasticity might have been underestimated. A study by Lange and Green (2004) found that respiration rates were able to fully acclimatise over a temperature range of 9 °C. As a result, the net carbon exchange and maximal net photosynthetic rates were very similar across seasonal changes in one year at their measuring site in Germany. The study measured six lichen species belonging to BSC, representing crustose, foliose and fruticose growth forms and having green algae and cyanobacteria as photobionts. This relatively flexible ability of the mycobiont to acclimatise would have implications for the ability of lichens to cope with a warming climate. Intraspecific genomic variability is not only relevant for the potential of evolutionary adaption, but can also impact gene expression responses to stress (Chavarria-Pizarro et al. 2022). Examples of (micro)climatic differentiation of mycobiont gene pools have been found, for example, by Nadyeina et al. (2014), Dal Grande et al. (2017), Belinchón et al. (2018) and Merges et al. (2023).

## Summary of the impacts and responses

The impacts of climate change are expected to be particularly pronounced in high latitude ecosystems, as the relative rise in temperatures is projected to increase polewards (IPCC 2023). Lichens have been shown to be sensitive to climate change, but responses appear species-specific and contradictory trends have also been reported (Chapin III et al. 1995; Molau and Alatalo 1998; Malmer et al. 2005; Jägerbrand et al. 2009; Hudson and Henry 2010; Lang et al. 2012; Bokhorst et al. 2016; Alatalo et al. 2017; Colesie et al. 2017; Götz et al. 2025). Additionally, species-specific responses may differ over time and short-term responses can be poor predictors of longer-term outcomes (Alatalo et al. 2015). Climate change affects lichens in various ways depending on factors including a species’ physiological responses to temperature and precipitation, dispersal capabilities, growth form, photobiont type, substrate preference and bacterial components of the symbiosis. Additionally, the evolutionary legacy of climatic niche evolution amongst the symbionts has been speculated to be important.

Climate change affects habitats in many ways. It increases the severity of storms and wildfires, causes sea level rise and affects snow cover. Climate change affects factors, such as temperature and moisture that are directly linked to growth and survival of the poikilohydric lichens. Some lichens can survive high heat and desiccation unharmed at 70–101 °C thallus temperature (Gasulla et al. 2021). However, in a hydrated and metabolically active state, lichens are more sensitive to heat stress, dying when the temperature exceeds 30–48 °C (Lange 1953; Chowaniec et al. 2023). Furthermore, the complex interactions of CO_2_, temperature and moisture mean that increased CO_2_ can either promote or inhibit lichen growth (Stanton et al. 2023). Theory suggests that as climate warms, the most dramatic effects would come from changes in species interactions and community assembly, instead of physiological tolerances (Urban et al. 2012; Blois et al. 2013; Cahill et al. 2014; Ockendon et al. 2014). Lichens have complex interactions with plants and animals and, therefore, phenomena, such as range shifts of tree species due to climate change, are becoming important for lichen communities.

Lichens are a combination of different taxa that together form a holobiont. Symbioses, such as lichens, are expected to be particularly susceptible to the effects of climate change, because climate sensitivity depends on the responses of individual symbionts, as well as coordination of the whole association – and the responses of the partners are often asymmetrical (Kiers et al. 2010; Colesie et al. 2017). Therefore, when estimating how lichens respond to climate change it is important to consider the different partners. The fungal partner may be sensitive, but its ability to adapt is also quite wide, at least in some cases (Lange and Green 2004). The photobionts, on the other hand, are diverse, including green algae (such as *Trentepohlia* and *Trebouxia*) and cyanobacteria. Lichens with *Trentepohlia* are more sensitive to frost than those with *Trebouxia*, but are able to utilise water vapour more efficiently (Nash et al. 1987). Photobiont performance and temperature sensitivity are speculated to be the basis for observed negative response in terms of species number and lichen cover under climate change in temperate and Mediterranean ecosystems (Aptroot and van Herk 2007; Escolar et al. 2012; Rubio-Salcedo et al. 2017; Smith et al. 2018). Alarmingly, a recent modelling study also shows that the photobionts of more than 7000 lichens could not adapt to temperature change as quickly as the Earth is warming (Nelsen et al. 2022). Contradictory to these trends, it has been implied that lichens with cyanobacteria possibly benefit from rising temperatures, because cyanobacteria possess a more efficient CO_2_ concentrating mechanism than green algae (Green et al. 1993). However, this topic is incompletely understood and significant reductions in nitrogenase activity at higher temperatures have also been reported (Antoine 2004; Liu et al. 2018).

Climate change causes distribution shifts that are already being observed in lichen communities. The quality of models predicting the distribution of future suitable climate space are fundamentally dependent on the accurate characterisation of the factors shaping current distributions. Factors such as biotic interactions, pollution, wind speed, irradiance and snow are important for species’ functional ecology and critical for developing more realistic predictions of how communities will shift under climate change (Ellis et al. 2007b; Mod et al. 2016; Ellis et al. 2017; Niittynen and Luoto 2018; Ellis 2019a). The adequate resolution of variables is especially important for lichens, as they are highly dependent on microclimatic factors, which are often overlooked.

As climate changes, lichens have three responses: acclimatisation, adaptation or extinction. Due to their symbiotic nature, one of the key questions for lichen acclimation and adaptation is whether the mycobiont can switch photobionts and which photobionts are available in future environments. Mycobionts are known to associate with several photobiont strains, although their level of specificity varies (Dal Forno et al. 2021; Pérez-Ortega et al. 2023). Furthermore, it is not clear yet if already established thalli could recruit new suitable symbiotic partners if needed. Some authors have argued that it would instead be essential to focus on distribution shifts and extinctions, rather than on evolutionary adaptations that cannot keep pace with the speed of recent climate change (Parmesan 2006; Jezkova and Wiens 2016; Smith et al. 2020; Nelsen et al. 2022).

## Conclusions and future directions

Although the impact of climate change has received attention from researchers for several decades (e.g. Insarov and Insarova (1996); Aptroot (2005); Ellis (2019a); Wrobleski et al. (2023)), it is difficult to identify universal responses for lichens. The response of lichens to human-induced climate also does not happen in a vacuum and must be interpreted in connection with other hazards such as pollution and habitat availability or quality (Binder and Ellis 2008; Svoboda et al. 2010; Ellis 2012; Ellis et al. 2014). Especially in large scale studies, it is challenging to separate abiotic and biotic effects, such as competition from vascular plants and land-use changes (Hauck 2009). All these factors might have compounding effects on lichens or interact in unexpected ways.

In lichenological research, there is a major bias towards high latitudes climates (polar, boreal, and temperate) and European and North American study sites. In addition to geographical bias, macrolichens are studied over other growth forms (Stanton et al. 2023), even though the diversity of crustose lichens is significantly higher. This is noteworthy because growth form may affect lichen responses to climate change (Rubio-Salcedo et al. 2017). Almost all ‘single species’ studies concern macrolichens, for example, *Evernia mesomorpha* (Smith et al. 2018; Meyer et al. 2023), *Flavoparmelia caperata* (Søchting 2004; Ellis 2019b), *Lobaria pindarensis* (Devkota et al. 2019), *Lobaria pulmonaria* (Nascimbene et al. 2016; Nascimbene et al. 2020; Di Nuzzo et al. 2022a; Bogale et al. 2024), *Nephromopsis pallescens* (Liang et al. 2012), *Usnea antarctica* (Bokhorst et al. 2016), *Ramalina maciformis* (Lange et al. 1977) and *Vulpicida pinastri* (Binder and Ellis 2008). Some notable exceptions do exist however, for example, *Myriolecis populicola* (Ellis et al. 2007) and *Protoparmeliopsis muralis* (Lange and Green 2008).

Yet another bias, as was pointed out by Canali et al. (2025), is that almost all available studies have only considered adult lichen thalli, causing a lack of knowledge on how a changing climate impacts the critical establishment phase.

Compared to plants and animals, fungi are less studied (e.g. Niskanen et al. (2023)). Lichenised fungi are a diverse group of organisms that are known from all terrestrial habitats. Over 20,000 lichen species are known (Lücking et al. 2017), but many more are unknown to science, with estimates reaching from 28,000 to more than 250,000 total known species (Sipman and Aptroot 2001; Lücking et al. 2009; Aptroot 2024). Even though research shows mainly adverse or even alarming effects of climate change on lichens, we do not yet have an overall understanding of how lichens are affected by climate change, because only a small fraction of lichen diversity has been investigated in this regard. Variation in responses may be natural, but we argue that contradictory trends amongst current studies exists partly because relatively few studies have been conducted and they are from a variety of locations, focus on different taxa and use varying methods. Therefore, it is hard to identify common patterns and draw general conclusions from the available data. Attempts to identify lichens that are particularly sensitive to climate change, either positively or negatively and which could therefore act as model or indicator species, are currently still unsatisfactory (Nelsen and Lumbsch 2020).

To improve our understanding on how climate change affects lichens, we recommend five steps. These approaches may be most effective in North America and Europe – where most of the facilities and background data exist – however, many of the recommendations can and should be utilised in other geographical areas as well:

1. Rapid climate change and the biodiversity crisis directs our focus towards methodologies that can “do more with less”, for example, towards using 'model species'. We recommend developing a set of model lichen species that represent lichen diversity, drawn from different geographical distributions, habitat types (incl. microhabitats), substrates, growth forms and photobiont types. In addition to studies on model species, we also call for studies on full communities. Although full community studies on lichens are time-consuming and require expertise, they are essential for understanding community interactions under a changing climate, amongst other things. Integrating results from model species to full community results would also be important for understanding the reliability and resolution of data.
2. Current resources and expertise are limited and, in comparison to other organism groups, lichens are often lacking wide historical and present-day records. This means that trying to merely apply methods from botany or zoology to lichenology may lead to unsatisfying results. To improve research data, we call for species monitoring programmes (including long-term monitoring/resampling where promising data are present) or integration in established programs (e.g. GLORIA network: https://www.gloria.ac.at), open data practices, active digitisation of observations and natural history collections in centralised accessible databases, such as GBIF and acknowledging trait data by including traits within collection digitisation.
3. Include a stronger recognition of developing technologies, such as transcriptomics which are being applied to understand lichen adaptation and stress responses (e.g. Chavarria-Pizarro et al. (2022); Fernandes Valim et al. (2025); Wong et al. (2025)).
4. Despite their ecological importance, lichens are often neglected in nature conservation planning. A recent summary of the 600 fungal Red List assessments showed that the largest fraction of newly-assessed lichens list climate change as an important threat, followed by development and human disturbance (Mueller et al. 2022; Yahr et al. 2024). However, the Red List evaluations of lichens are often based on deficient data due to limited resources and observations. Globally only 145 lichen species have been assessed for the IUCN Red List, which is 0.73% of the currently known 20,000 lichen species. Assessments are also conducted nationally, but with varying comprehensiveness and at different scales. The lack of knowledge of lichen species is especially true in the Tropics, even though these areas are often megadiverse in their lichen flora/funga (Yahr et al. 2024). This means that even dramatic decreases or extinction of rare and unknown species might go unnoticed. We suggest that evaluations could try to utilise data that go beyond single species boundaries, as well as evaluating the state of species communities and their habitats, for example, by seeking evidence for the impacts of threats to species that are ecologically similar and closely-related and occur in specific regions (also suggested, for example, by: Yahr et al. (2024); Goodsell et al. (2025)).
5. Finally, lichens are not simply symbioses of the mycobiont and photosynthesising partner the way was previously thought. Instead, lichens are small ecosystems and a combination of several partners that may be metabolically linked. We need to acknowledge that climate change may affect the partners in ways that we do not yet know or understand.

## Additional information

### Use of AI

No use of AI was reported.

### Author ORCIDs

Lilith Weber https://orcid.org/0000-0002-6856-8095

Pekka Niittynen https://orcid.org/0000-0002-7290-029X

Annina Kantelinen https://orcid.org/0000-0001-8664-7662

### Data availability

All of the data that support the findings of this study are available in the main text.
